# POCUS and POCDUS: essential tools for the evaluation and management of carotid artery pseudoaneurysms after a gunshot wound

**DOI:** 10.1186/s13089-020-00182-7

**Published:** 2020-07-22

**Authors:** Lisandro Montorfano, Marianna Sarkissyan, Matthew Wolfers, Federico Rodríguez, Fernando Pla, Miguel Montorfano

**Affiliations:** 1 Department of General Surgery, Cleveland Clinic Florida, Weston, FL USA; 2 Department of Ultrasound and Vascular Doppler, Hospital de Emergencias “Dr. Clemente Alvarez”, Av. Pellegrini 3205, Rosario, Santa Fe Argentina

**Keywords:** Point-of-care ultrasound, Point-of-care Doppler ultrasound, Doppler ultrasound, Penetrating trauma, Vascular injury, Carotid injury, Pseudoaneurysm, POCUS, POCDUS

## Abstract

**Background:**

Evaluation of asymptomatic penetrating vascular injuries can be done with Point-of-care ultrasound (POCUS) and Point-of-care Doppler ultrasound (POCDUS).

**Case presentation:**

A 21-year-old woman was admitted to the Emergency Department with a small wound and pain on the left side of her neck. The patient stated she was standing outside her home and suddenly felt acute pain in the neck. She denied trauma or being assaulted and reported no significant past medical or surgical history. On physical exam the only positive finding was a small gunshot entry wound on the left side of her neck without hard signs of vascular injury. Bedside POCUS demonstrated soft tissue swelling and a hematoma next to the left carotid artery. A round in shape bullet was visualized in contact with the posterior left common carotid artery wall and two small saccular pseudoaneurysms were seen at left common carotid artery wall. POCDUS showed a patent left carotid artery and turbulent flow in the two saccular aneurysms. A computed tomography angiogram (CTA) was performed confirming the findings and a stent in left carotid artery was placed. The patient tolerated the procedure well and was discharged 4 days after the procedure. At the sixth month follow-up, Doppler ultrasound showed patent stent and resolution of the muscular hematoma.

**Conclusions:**

Penetrating trauma-related vascular injuries are complex cases to handle within an acute setting. POCUS and POCDUS are increasingly being used for the workup and decision-making process of gunshot-related vascular injuries to the neck and are a fundamental part of the follow-up after definitive therapy.

## Background

Violence and crime in Latin America have been considered “the social pandemic of the 20th century” [1]. Traumatic vascular injuries represent approximately 3% of all traumatic injuries. The majority of these lesions are caused by gunshot wounds, stab wounds, and blast injuries or from penetrating debris [2]. Mortality from high-velocity traumatic injuries, such as gunshot wounds, specifically to the neck, is estimated to be as high as 5%. Hence, expedited diagnosis and treatment are essential to reduce disabilities and to prevent death [2–7]. When patients present with hard signs of vascular injury (such as external bleeding, expanding hematoma, absent distal pulses, palpable thrill, and audible bruit) surgical exploration is mandatory. When soft signs or no clear signs of vascular injury are present and the patient is hemodynamically stable, vascular injuries present a challenge.

Traditionally, the approach to treatment of penetrating vascular injuries to the neck has been guided by damage to the anatomic zone [8, 9]. In brief, Zone III spans the neck from base of skull to the angle of mandible, Zone II from the angle of mandible to cricoid cartilage, and Zone I from cricoid cartilage to the thoracic inlet. Injury to Zone II is most common, and injury to Zone I is more life-threatening and traditionally most likely to require immediate surgical exploration. Penetrating neck injuries can involve laryngotracheal anatomy resulting in respiratory distress and symptoms, pharyngoesophageal injuries that can affect the aerodigestive functions, or involve the nervous system injuries, which can impact autonomic and even spinal functions. Vascular injuries are the most common cause of immediate mortality due to exsanguination, with the highest rate related to carotid artery injury [9].

Imaging can be useful in the diagnosis, management, and follow-up of vascular injuries [3, 10, 11]. Angiographic evaluation of these injuries continues to be the gold standard; however, noninvasive modalities such as CTA, magnetic resonance angiography, and Doppler ultrasound represent valid alternatives [11–17].

Point-of-care ultrasound (POCUS) and Point-of-care Doppler ultrasound (POCDUS) are cheaper, noninvasive and faster alternatives to diagnose vascular injuries [14, 15]. Bergstein et al. found that, using arteriography as the gold standard, Color Flow Doppler (CFD) had a specificity of 99%, sensitivity of 50%, and Fry et al. described 100% sensitivity and specificity of Doppler ultrasound compared with the conventional arteriography and operative exploration [12, 13]. In our previous study using a FAST Doppler examination (FAST D protocol), we found 100% sensitivity and specificity to rule out vascular injury in penetrating gunshot wounds of the lower limbs [15]. In many facilities, POCUS and POCDUS are the first diagnostic modality available, and are used for a whole body examination, from head to toe, allowing for an integrated management of the trauma patient [14–16]. Moreover, in some rural or remote places, those are the only diagnostic resource available. The aim of this study is to describe POCUS and POCDUS characteristics of an asymptomatic penetrating vascular injury to the neck after a gunshot wound.

## Case report

**Fig. 1:**
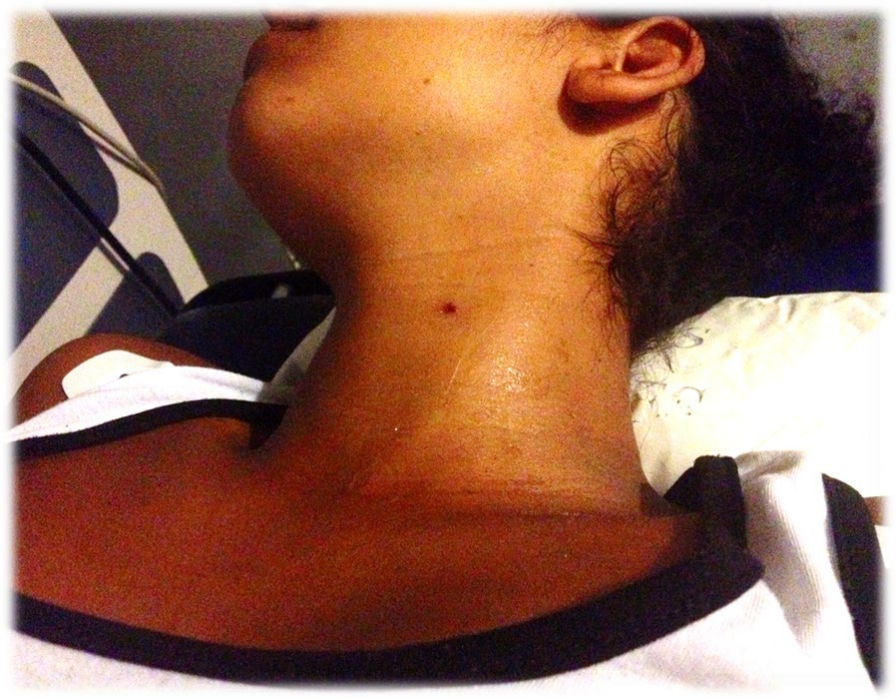
Gunshot wound to the neck

A 21-year-old woman was admitted to the Emergency Department with neck pain and a small wound on the left side of her neck (Fig. 1). The patient stated she was standing outside her home and suddenly felt acute pain in the left side of the neck. She denied trauma or being assaulted at the time of the occurrence and reported no significant past medical or surgical history. On physical exam, the patient was in no acute distress, and vital signs were stable and within normal limits. The only positive physical exam finding was a small gunshot entry wound on the left side of her neck without hard signs of vascular injury.

**Fig. 2:**
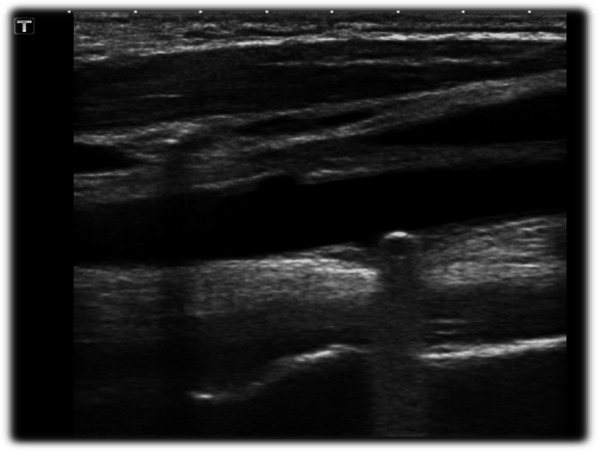
Ultrasound view of left common carotid artery demonstrating two saccular pseudoaneurysms and bullet

**Fig. 3:**
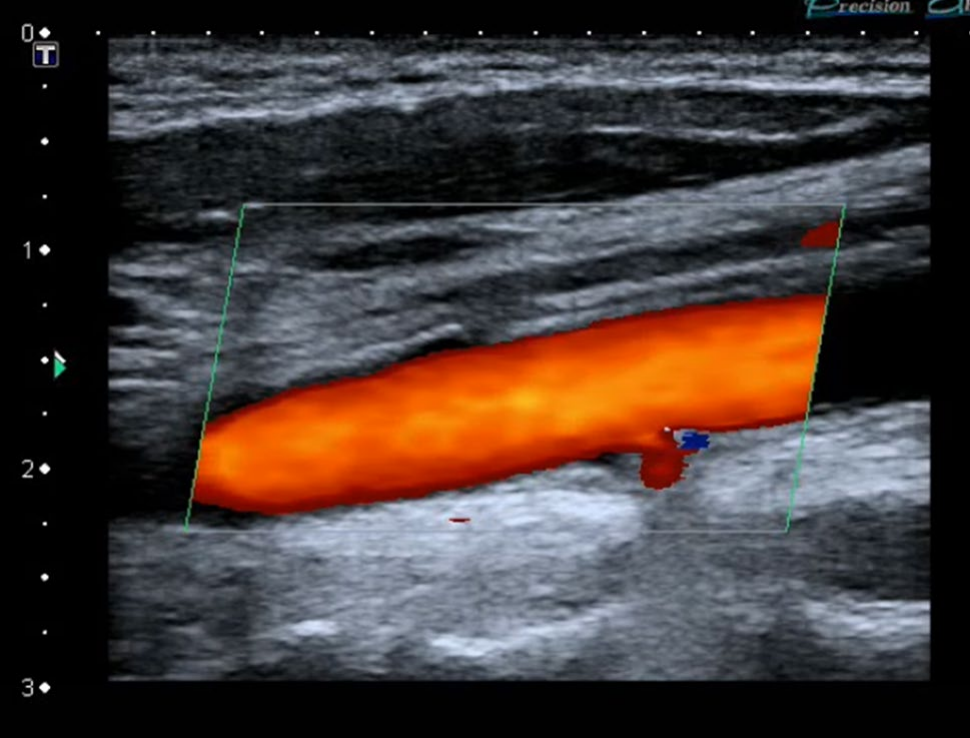
Color Doppler Imaging of carotid demonstrating turbulent flow in the posterior wall pseudoaneyrysm

**Fig. 4:**
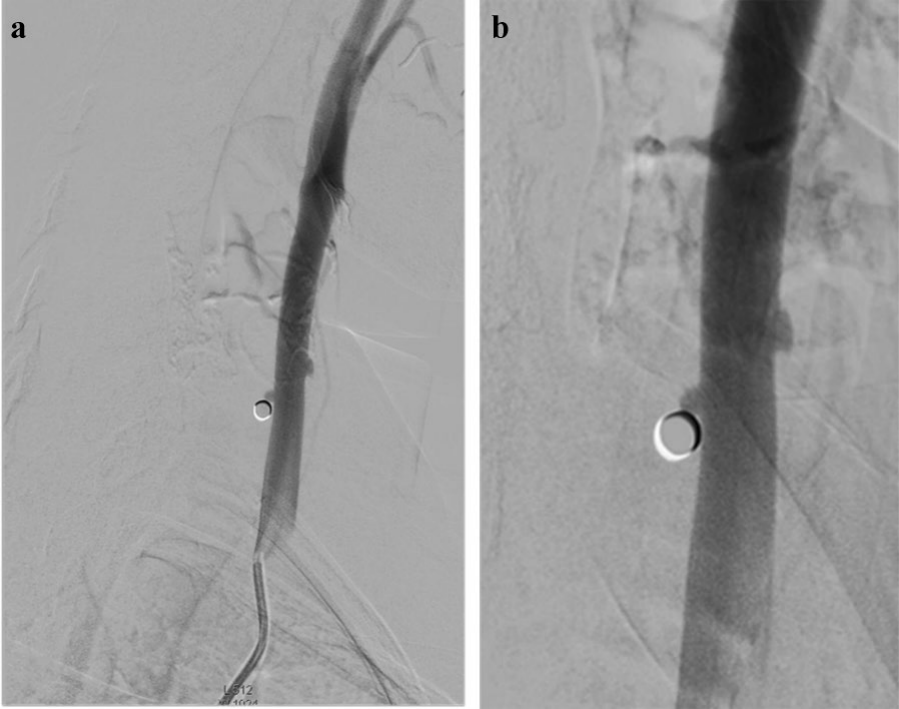
**a**, **b** Views of angiography of the left carotid artery showing the two pseudoaneurysms and bullet

**Fig. 5:**
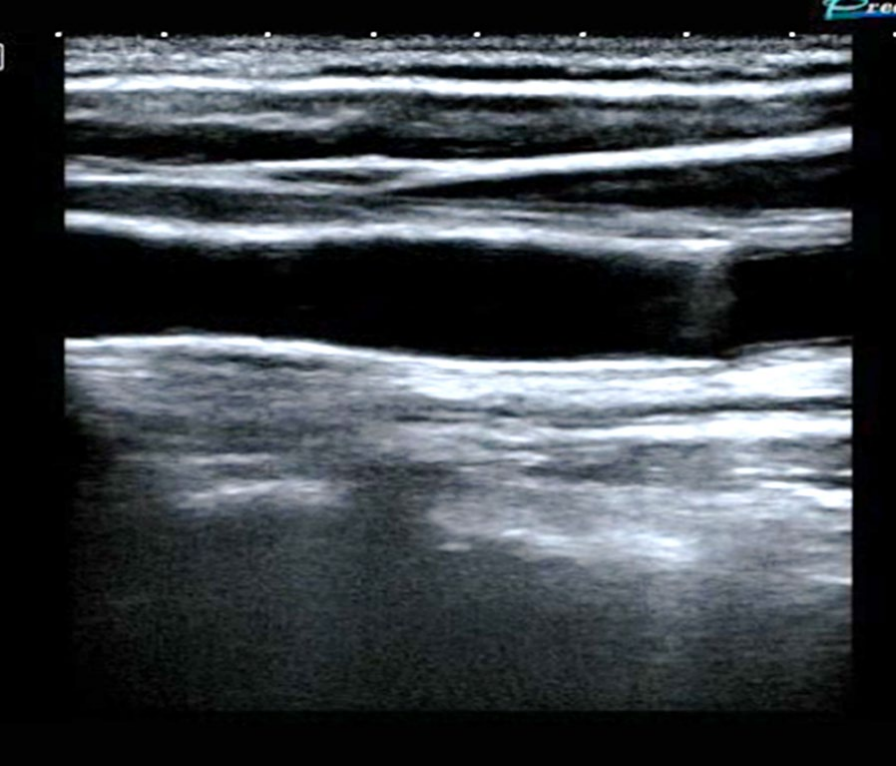
Ultrasound of carotid after stenting: Longitudinal view

**Fig. 6:**
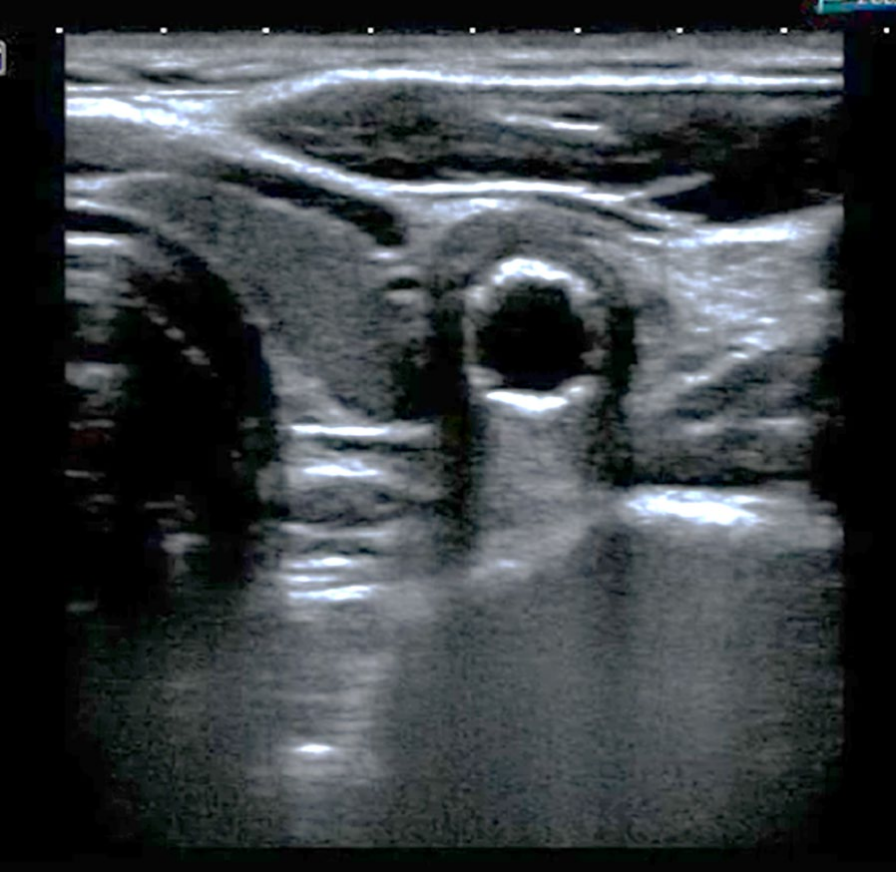
Ultrasound of carotid after stenting: Transverse view

**Fig. 7:**
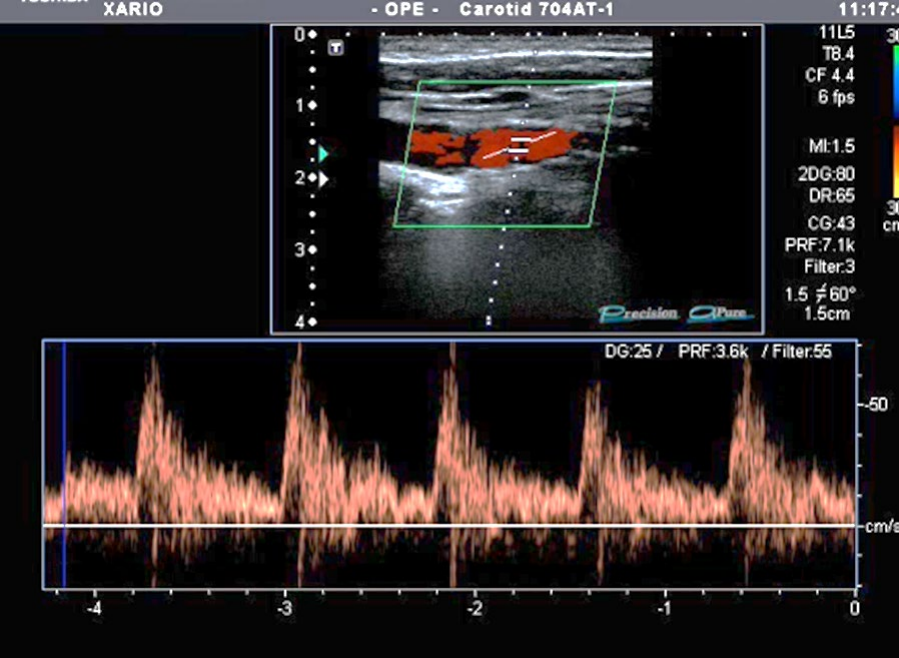
Color and pulsed Doppler demonstrating restored normal flow in common carotid artery at the site of the stent

Bedside POCUS was performed and demonstrated soft tissue swelling and a hematoma next to the left carotid artery. A round in shape bullet, with metallic like reverberation artifact, was visualized in contact with the posterior left common carotid artery wall. Two small saccular pseudoaneurysms were seen at left common carotid artery wall (Fig. 2). POCDUS showed a patent left carotid artery and turbulent flow in the two saccular aneurysms (Fig. 3). The studies were performed with a broadband linear array transducer with a frequency range of 5–12 MHz (Toshiba Xario; Tokyo, Japan). Patient was scanned in a supine position. Transverse and longitudinal scans of the vessel were performed. Due to the findings on ultrasound and color Doppler, a computer tomography angiogram was performed. The CTA confirmed these findings (Figs. 4a, b) and the decision was made to consult interventional radiology. Interventional radiology placed a left carotid stent (Figs. 5, 6, 7) and anti-platelet aggregators were given. The patient tolerated the procedure well and was discharged 4 days after the procedure. At the sixth month follow-up, Doppler ultrasound showed patent stent and resolution of the muscular hematoma next to the left carotid artery.

## Discussion

Penetrating trauma-related vascular injuries are challenging. When patients present with hard signs of vascular injury such as hemorrhage or expanding hematomas, surgical exploration is mandatory [9]. When soft signs or no clear signs of vascular injury are present and the patient is hemodynamically stable, imaging methods could be useful tools in the diagnosis and management of these patients. CTA continues to be the gold standard for the diagnosis of these injuries. However, POCUS and POCDUS are a cheaper, noninvasive and faster alternative to diagnose these injuries. As described in this manuscript, these tools can provide valuable information at the bedside and guide the decision-making process and follow-up of these patients.

High-frequency linear probes are the probes of choice because higher resolution and lower penetration are needed to assess this kind of injuries. When evaluating a penetrating vascular injury, it is important to scan the region of the trauma in a transverse and longitudinal fashion with B-mode. Flows should be investigated with Color and Duplex Doppler ultrasound. For comparative purposes, it is advised to evaluate both sides of the neck—affected and unaffected sides. Veins in the neck are oval in shape and are compressible. The Duplex Doppler evaluation shows a continuous flow pattern with respiratory variations. Arteries are round in shape and non-compressible. Doppler ultrasound is a useful tool for the assessment of anatomy and flow dynamics of the carotid arteries. The Duplex Doppler evaluation shows a pulsatile flow pattern with low resistance in the common and internal carotid artery and high resistance in external carotid artery.

Large hematomas, subcutaneous air, morbid obesity or large open wounds can represent a challenge, yet several studies have shown that POCUS and Doppler ultrasound remain useful tools to rule in or rule out trauma-related vascular injuries [11, 12, 14, 15]. As described in a previous paper, in patients with clinical suspicion of a vascular injury, POCUS and POCDUS are used to answer 2 basic questions [15]:

1. Does the patient have a vascular lesion? Yes or no?
2. If the answer is yes: What kind of lesion?

Two pseudoaneurysms of the left common carotid artery were found in this patient. A pseudoaneurysm is the result of a partial rupture of an artery wall, which causes accumulation of periarterial blood. The accumulated blood is surrounded and contained by the adventitia or adjacent hematomas or soft tissues. Traumatic carotid injuries are divided into grades, which can assist in guiding management [18]. Grade I injuries are mild intimal injuries which do no cause significant hemodynamic injury, grade II involve dissections and hematomas which do impact vascular hemodynamics, grade III are pseudoaneurysms (such as in this case), and grade IV and V are occlusions and transection with extravasation, respectively [18]. In this case report, the small pseudoaneurysms could have been caused by direct hit or by the heat the bullet generated next to the artery. Pseudoaneurysms always communicate with the arterial lumen by the pseudoaneurysm neck. While penetrating trauma is a common cause [19, 20], the most common cause is iatrogenic pseudoaneurysm [11, 21].

Evaluation of pseudoaneurysms with Doppler ultrasound on B-mode will demonstrate an anechoic or hypoechoic image containing moving echoes. It may increase in size during systole. The presence of a partially filled lumen by echogenic structures may represent a thrombus. The color flow evaluation will demonstrate the typical swirling motion known as the Yin–Yang sign, which is caused by the circular motion of the blood inside the pseudoaneurysm. During systole, the flow goes toward the pseudoaneurysm and during diastole the blood moves back to the arterial lumen [20, 21]. Spectral analysis will demonstrate turbulent flow. The pulse Doppler with the sample volume placed at the level of the neck of the pseudoaneurysm will show a flow pattern known as “to-and-fro”, a bidirectional flow caused by the continuous entry and exit of blood from the artery to the pseudoaneurysm and vice versa.

The management of traumatic carotid injuries are guided by grade and appropriate and timely interventions are warranted to prevent sequelae, such as stroke and occlusions, causing permanent central nervous system-related morbidity and mortality [18, 22–24]. Traditionally, grade I and II have been successfully managed with anticoagulants or anti-platelet equivalents (if there are no contraindications precluding its use). Among patients being managed with anti-coagulant therapy, PT/INR ratio should be maintained between 2 and 3, for 3–6 months [18, 22–24]. Unlike grade I–II injuries, however, grade III–V injuries require invasive therapies, either endovascular or surgical.

Grade III blunt carotid injuries have a stroke rate as high as 33% and mortality up to 11% [18, 24] with grade IV and V having even higher risks. Hence, timely and effective treatment often combining anticoagulation with procedural intervention is advisable. Endovascular interventions such as stenting present reasonable alternatives to open surgical techniques. A review by Morr et al. evaluated various stenting interventions to traditional open surgery methods such as endarterectomy [25]. Many factors of the stent characteristics themselves, including bare-metal vs. covered, tapered vs. untampered, and drug eluting vs. cutting/balloon based are pertinent to the discussion. Overall, stenting is a safe alternative to traditional surgical techniques such as carotid endarterectomies in some situations. Complications from endovascular procedures around stenting have been limited to few cases of carotid artery spasm, which can be medically managed with nitrates or calcium channel blockers.

A meta-analysis by Pham et al. evaluated the utility of endovascular stenting in carotid pathologies and included 31 studies with over 140 patients. The review showed that 98.4% of pseudoaneurysm cases were either successfully stented or occluded when appropriate [26]. Endovascular stenting is a safe and appropriate alternative to surgical intervention but randomized clinical trials are still needed. Maras et al. also reviewed the safety and short-term efficacy of covered stents for traumatic extracranial carotid artery pseudoaneurysms [27]. The review included 20 patients with carotid pseudoaneurysms secondary to traumatic injury and demonstrates that endovascular approaches can spare the morbidity and risk of surgical repair while providing similar efficacy in maintaining patency. Notably, endovascular approaches are less likely to result in cranial nerve injuries which are associated with open surgical approaches. While together these reviews and studies suggest a strong role for stenting in grade III blunt carotid injuries, longer follow-up on more patients is needed to further examine this promising treatment in addition to randomized clinical trials.

## Conclusions

POCUS and POCDUS are widely available, noninvasive, sensible and specific techniques that are increasingly used for the diagnosis and decision-making process of penetrating vascular injuries to the neck and are a fundamental part of the follow-up after definitive therapy.

## Data Availability

Not applicable.
